# Effect of the physicochemical changes in the antimicrobial durability of green synthesized silver nanoparticles during their long-term storage†

**DOI:** 10.1039/d2ra04667a

**Published:** 2022-10-25

**Authors:** Giyaullah Habibullah, Jitka Viktorova, Pavel Ulbrich, Tomas Ruml

**Affiliations:** Department of Biochemistry and Microbiology, University of Chemistry and Technology Technická 3, 166 28 Prague Czech Republic habibuli@vscht.cz

## Abstract

It is generally recognized that the stability of nanoparticles (NPs) has a great impact on their potential biological applications. Despite this, very few studies have investigated the change in toxicity of NPs over time but none has studied the periodic physicochemical changes contributing to it. To address this, we analyzed the effects of long-term storage on the physicochemical changes of green synthesized silver nanoparticles (AgNPs) that directly influences their antimicrobial durability. Light-induced slow synthesis of AgNPs was carried out using *Saraca asoca* aqueous leaf extract. The synthesis was optimized with respect to parameters known to play a major role in the long-term stability of AgNPs: pH, temperature, light exposure time, AgNO_3_ concentration, extract proportion in the reaction mixture and storage conditions. Freshly synthesized AgNPs were characterized and then stored under optimized conditions. UV-vis spectrophotometry, AAS, conventional TEM and HR-TEM along with EDX spectroscopy were used at regular intervals to test the physicochemical properties that influence their long-term stability. Broth dilution assay was used to test antimicrobial activity of AgNPs against *Escherichia coli* and *Staphylococcus aureus*. Under dark storage conditions at room temperature, the AgNPs exhibited excellent stability with very good dispersity, throughout the study period of 18 months, despite the particles undergoing physicochemical changes in largescale. AgNPs exhibited sufficient antimicrobial activity against both strains tested. Due to the stronger stabilizing effect of the extract, we observed the lowest inhibition of *E. coli* and *S. aureus* by the freshly synthesized and 15 day old AgNPs; however, the inhibition rate escalated after a month and the highest rate of inhibition was observed with the particles between 2 months to 6 months of storage. After 6 months, we observed the particles losing their antimicrobial potential gradually, that lasted throughout the rest of our study period. This observation was in accord with the physicochemical changes that AgNPs were undergoing with time. By deepening our understanding of the changes in the physicochemical properties of green synthesized AgNPs over time, this study contributes to the development of more effective, durable, and potent AgNPs.

## Abstract 2

A new method for light induced green synthesis of silver nanoparticles that supports long-term stability. Elevation of oxidation and dissolution levels with time directly impacts the long-term stability and antimicrobial durability.

## Graphical abstract

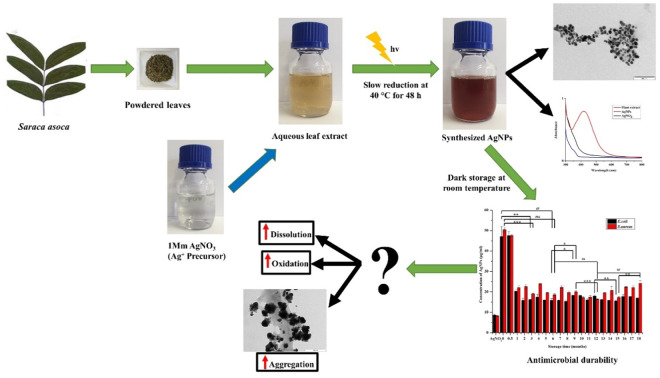

## 1 Introduction

Cross-disciplinary nanoscience research involving chemists, physicists, biologists, and engineers aims to develop eco-friendly and sustainable methods for the synthesis of nanoparticles. Most of the classical methods used for nanoparticle (NP) preparation are sophisticated and expensive, require skilled labor, and often involve the use of toxic and hazardous chemicals. The latter poses potential biological risks, as noble metal NPs can come into contact with human epithelium because they are becoming an integral part of everyday life (in therapeutics, cosmetics, biosensing, diagnosis and medicine).^1–5^ Current efforts to address this concern are focused on integrating green chemistry approaches into the design of environmentally safe and sustainable materials and processes.^6,7^

Silver (Ag), in the form of salts, colloids and the metal itself, has long been employed in the treatment of chronic infections and as an antimicrobial agent. The first historical evidence of the application of silver in therapeutics has been dated back to 1500 BC by the Han Dynasty, China.^8,9^ The ability of silver nanoparticles (AgNPs) to exhibit antimicrobial activity at very low concentrations that are non-toxic to normal mammalian cells,^10^ has attracted intensive research interest in the recent years. Consequently, AgNPs now have important applications in therapeutics, diagnostics and catalysis, as well as in analytical techniques as signal enhancer in surface-enhanced Raman spectroscopy (SERS).^11–13^ In recent years, there has been an upsurge of interest in the potential use of AgNPs to combat antibiotic resistance, which is often caused by the inappropriate/overuse use of antibiotics.^14,15^ The synergistic effect exerted by AgNPs when coupled with antibacterial drug groups, such as β-lactams,^16^ aminoglycosides, glycopeptides and sulfonamides,^17^ enhances antimicrobial activity against multidrug-resistant bacteria. This ability makes AgNPs an ideal candidate for treating some chronic infections caused by microorganisms.

Despite all these advantages, AgNPs suffer from a major drawback that limits their range of application – lack of long-term stability. The physicochemical changes (oxidation, ion release, agglomeration, aggregation) that take place in chemically synthesized AgNPs during their long-term storage play a vital role in altering their toxicity over time.^18,19^ Their long-term stability depends on various factors, including the method of synthesis and storage conditions, but the choice of stabilizing agent introduced after/during synthesis is also crucial. Many of the stabilizers currently used (*e.g.*, sodium citrate, sodium dodecyl sulfate, chitosan) have the tendency to alter the charge of the particles and maintain the repulsive force between them for a long time when stored under constant storage conditions thus preventing the aggregation of the particles. Over the time, the repulsive forces between the particles decrease due to slow degradation of the stabilizing agent on the surface of the NPs, thereby resulting in their excessive oxidation and dissolution causing aggregation and agglomeration.^20–22^ The AgNPs stabilized with high molecular weight stabilizers (polymers, Tween 80® and Disperbyk-129) have proved to be more stable than other commonly used stabilizers.^19,22^ These stabilizers completely engulf the particles, resulting in much less ion release and oxidation regardless of the influence of storage conditions on long-term stability. However, the high molecular weight stabilizers increase the hydrodynamic size as well as the polydispersity index (PDI) of the system, which leads to the higher risk of aggregation. Generally, these stabilizers at higher concentration become toxic^23^ and also block the ion release from NPs, thereby, limiting their biological applications (especially antimicrobial ability). To address the problem of the stabilizing agent affecting the long-term stability and biological applications of NPs, researchers have begun to investigate a green chemistry approach that involves the use of plant leaf extracts in AgNPs preparation.^1,24^ The main advantage of this approach is that the ability of plant extract components to serve as reducing and stabilizing agents eliminating the need to introduce a stabilizing agent into the system after NPs synthesis.^7,25^

In recent years, there has been an upsurge in the research interest of green synthesized AgNPs. This green synthesis not only employs plant component extract but also uses culture medium filtrate and the microorganisms (both bacteria and fungi), as a result, the number of reports published also increased (especially in case of AgNPs).^11,26^ Proving, the ultimatum of green synthetic methods which is the ease of synthesis, that leads to the elimination of most of the issues listed in the synthesis of NPs above. In these reports most attention has been paid to the exploration of new substrates and optimization of reaction parameters that would support the effective synthesis of NPs. However, the second ultimate target of using green synthesis is yet to be fully achieved, which is stability of NPs especially in long-term.

Although the long-term stability of physically and chemically synthesized AgNPs (Table 1) has been the subject of intensive studies, similarly, the primary focus has been on optimizing synthesis reaction parameters and storage conditions, with much less attention devoted to the physicochemical and toxicity changes that occur in AgNPs over time.

**Table 1:** Reports that studied long-term stability of AgNPs

Source of AgNPs synthesis	Size range (nm)	Storage conditions	Stability	Periodic evaluation	Main physicochemical method used	Application	Periodic application evaluation	Ref.
*Cassia roxburghii* DC (leaf aqueous extract)	10–30	Dark, room temperature	12 months	Yes	UV-vis	Antifungal	No	1
LASER ablation	10–70	Both light and dark, room temperature and 4 °C (only dark)	13.5 months	No (only initial and final measurements)	STEM coupled with EDX spectroscopy and mapping	Antibacterial	Yes (every 14 and 45 days)	18
*Parachlorella kessleri* (whole aqueous extract)	5–30	Both light and dark, room temperature and 5 °C (only dark)	6.6 months	Yes	UV-vis and TEM (size analysis)	No	No	27
Chemical reduction of AgNO_3_ with NaBH_4_ in the presence of Na_3_C_6_H_5_O_7_	2–10	Dark, room temperature and 4 °C	12 months	No (only initial and final measurements)	UV-vis and TEM (size analysis)	No	No	21
Chemical reduction of AgNO_3_ with NaBH_4_ in the presence of different stabilizing agents	Not disclosed	Both light and dark, room temperature and 4 °C (only dark)	6 months	No (only initial and final measurements)	UV-vis, zeta potential, ICP-MS, DLS, XRD and TEM (size analysis)	Cytotoxic studies	No (only initial and final measurements)	19
Chemical reduction of AgNO_3_ with NaBH_4_ in the presence of different stabilizing agents	Not disclosed	Dark, room temperature	6 months	No (only initial and final measurements)	UV-vis, zeta potential and DLS	Detection of Mn^2+^ ions	No	28
*Annona muricata* (ethanoic extract)	2–500	Dark, room temperature and cold storage (4, −20 and −80 °C)	3 months	No (only final)	UV-vis	No	No	29
*Derris trifoliata* seeds (aqueous extract)	Not disclosed	Dark, room temperature	>3 months	No	Not disclosed	Antioxidant, antibacterial and antiproliferative	No	30
*Juglans regia* (aqueous extract)	Not disclosed	Dark, room temperature and 4 °C	6 months	No	Not disclosed	Antioxidant, antimicrobial, cytotoxicity	No	31
Chemical reduction using pectin	8–28	Dark, 4 °C	5 months	Yes	UV-vis	Antibacterial	No	32
*Sargassum angustifolium* aqueous extract along with chitosan	5–20	Dark room temperature	6 months	No (only initial and final measurements)	UV-vis	Antibacterial	No	33

Even though, green synthesized AgNPs has outperformed chemically synthesized NPs in biological applications and short-term stability (Table 1),^34,35^ still the studies exploring the stability in long-term has not gained enough research attention as in case of physical and chemical methods. Consequently, very few researchers have reported the long-term stability of AgNPs synthesized by using biological sources (green synthesis),^1,27^ but none evaluated the change in toxicity and physicochemical properties of AgNPs over time. To address this, we tested over 18 months the antimicrobial durability of AgNPs synthesized from *Saraca asoca*'s (Roxb.) aqueous leaf extract by light-induced slow synthesis method. The physicochemical changes taking place in the AgNPs were also monitored during synthesis and at regular intervals during their long-term storage.

## 2 Materials and methods

### 2.1 Chemicals

Following analytical grade chemicals were purchased from: silver nitrate, Fluka, Czech Republic; Mueller–Hinton (MH) broth; Sigma Aldrich, USA. Bacterial cultures (*Escherichia coli*, CCM 4517, *Staphylococcus aureus*, DBM 7002) were obtained from the culture collection of Department of biochemistry and microbiology (DBM), UCT, Prague, and Czech collection of microorganisms (CCM), Brno, Czech Republic.

### 2.2 Collection of plant materials

Healthy and matured leaves of *Saraca asoca* were collected in June 2018 from the garden of Government maternity hospital, Chidambaram, India (11.3982° N, 79.6954° E). Identification of the plant was performed by the Department of botany, Annamalai university, Annamalai Nagar, Chidambaram, India (accession no: 358). The collected leaves were washed, and shadow dried for 10 days at room temperature. The dried leaves were coarsely powdered, air tightly packed and stored at room temperature (20 ± 3 °C) for further work.

#### 2.2.1 Preparation of extracts

Four grams of the dried leaf powdered sample were mixed with 100 ml of sterile Millipore water. The mixture (40 g l^−1^) was heated at 70 °C for about 30 min in a heating mantle. The mixture was cooled and then filtered with Whatman filter paper No. 1. The filtered extract was stored in refrigerator (4 °C) for future use.

#### 2.2.2 Optimization of silver nanoparticles production

Optimization of AgNPs synthesis was performed by one variable at a time method (OVAT)^36^ considering the parameters that play a vital role in effective synthesis and stability of AgNPs: effect of light in synthesis and storage, extract volume, AgNO_3_ concentration, temperature, and pH. Volume ratio of 9 ml of 1 mM AgNO_3_ solution to 1 ml of aqueous extract of *S. asoca* (40 g l^−1^) (10% (v/v)) and monitoring time of 48 h was applied throughout the optimization studies, except while studying the effect of extract volume in AgNPs synthesis. Simultaneously, aqueous leaf extract without the addition of AgNO_3_ was used as control. To determine the effect of illumination in the synthesis and storage of AgNPs, the reaction mixture was maintained in dark (0 lx) at room temperature (20 ± 3 °C). At the same time, another sample set was exposed to fluorescent tube light (1140 lx) at room temperature. After the synthesis, the reduced mixture (after 48 h) was kept under normal laboratory conditions (180 lx ambient laboratory light, 20 ± 3 °C) for 15 more days to study the effect of light during storage of AgNPs. Concurrently, parameters such as different AgNO_3_ concentration ranging from 1 to 10 mM (at a constant extract volume), pH ranging from pH 2 to 12 and different temperatures ranging from 20 to 90 °C were also tested for synthesis yield and stability of AgNPs. The pH values were adjusted using 0.1 M HCl or 0.1 M KOH solutions. Different volume percent of *S. asoca* leaf extracts (40 g l^−1^) such as 5, 10, 15, 20, 25, 30, 35, 40, 45, 50% (v/v) in different salt concentrations of AgNO_3_ (namely 1, 3, 5, 7, 10) were also optimized for the synthesis of stable AgNPs. As the reaction results in color change, absorbance of the resulting solution was measured spectrophotometrically in the range of 300–800 nm.

### 2.3 Synthesis of silver nanoparticles (AgNP_S_)

The synthesis procedure was as follows: to 90 ml of 1 mM AgNO_3_ solution, 10 ml of aqueous leaf extract of *S. asoca* (40 g l^−1^) was added and illuminated (fluorescent tube light, 1140 lx) for 48 h at 40 °C. After reduction, the mixture was stored in dark at room temperature (20 ± 3 °C). A portion of the reduced mixture was then centrifuged twice at 20 000×*g* for 30 min to eliminate residual soluble compounds and the pellets obtained were resuspended in 5 ml of sterile Millipore water for further characterization. A part of the supernatant was treated with 2 M HNO_3_ solution (1 : 4 ratio) for atomic absorption spectroscopy (AAS, AGILENT 280 FS AA SPECTROMETER, Agilent Technologies, Australia) to determine the silver ion (Ag^+^) concentration in the reduced mixture. The same procedure was followed at regular time intervals (48 h, 7th day, 15th day, 1 month, 3 months, 6 months, 12 months, and 18 months) to study the dissolution of AgNPs during their long-term storage.

### 2.4 Characterization of silver nanoparticles

Primary characterization of AgNPs started with visual examination of color change (from golden yellow to reddish brown) followed by absorption spectrum scanning at a wavelength range of 300–800 nm (SpectraMax i3x Multi-Mode Detection Platform, Molecular Devices, USA). The color change being a typical indicator of AgNPs synthesis, was also considered as a crucial indicator of the AgNPs stability, as the nanoparticles age the brown color of the system becomes more intense and accompanied by sedimentation in case of particles aggregation.^1,27^ Stability of AgNPs was monitored by sampling the reaction mixture at time intervals of 24 h, 48 h, 7 days, 15 days, 1 month, 3 months, 6 months, 10 months, 12 months, and 18 months. For electron microscopic studies, 10 μl of synthesized AgNPs was placed on a carbon coated copper grid (prepared grids were vacuum dried HR-TEM in order to limit the interference of the atmospheric oxygen in EDX measurement) and the images of NPs were collected using conventional TEM (JEOL 1010, JEOL, Japan) and high resolution transmission electron microscopy (HR-TEM) equipped with selected area electron diffraction (SAED) and energy dispersive X-ray analysis (EDX) (JEOL 2200FS, JEOL, USA). Periodic electron microscopy along with EDX analysis (48 h, 1 month, 3 months, 6 months, 12 months, and 18 months) of AgNPs was performed to track the changes in their physicochemical properties (oxidation, agglomeration, and aggregation) during their long-term storage. Freshly synthesized AgNPs were lyophilized (Freezone 2.5, Labconco, USA), and a part of the sample was mixed with KBr in the ratio of 1 : 10 and casted in the form of pellets for Fourier transform infrared spectroscopic analysis (FTIR) (Nicolet 6700, Thermo-Nicolet, USA) with the spectral range of 400–4000 cm^−1^.

### 2.5 Antimicrobial activity of NPs

Concentration halving the population viability (IC_50_) and minimal inhibition concentration (MIC) of the AgNPs was determined by microplate broth dilution method. The overnight cultures of *E. coli* and *S. aureus* were diluted to achieve an inocula with turbidity equal to 0.5 McFarland unit. An aliquot of 100 μl from this inoculum was added to each well of a 96-well microtiter plate and 100 μl of the test sample (108 μg ml^−1^) was added only to the first column and two-fold progressive dilutions of the sample as well as the control was prepared. After a minute of intense shaking, the initial absorbance was determined at 600 nm with Gen5 spectrophotometer (BioTek, USA). The plates were then incubated at 37 °C overnight and the final absorbance was also recorded. IC_50_ was calculated from the difference of final and initial absorbance with the freely available IC_50_ calculator (AAT Bioquest, USA). MIC was evaluated by visual examination and recorded as the lowest concentration that completely inhibited the growth. We applied periodic triplicate testing at the regular time intervals of 30 days up to 18 months for monitoring the change in AgNPs antimicrobial durability over the time.

### 2.6 Statistical analysis

Statistical significance of the results was tested by one-way ANOVA. For all statistical tests, the significance level of *p* < 0.05 was established. The values presented are the mean of replicas ± standard error of the mean (SEM).

## 3 Results and discussion

### 3.1 Synthesis of AgNPs from *S. asoca* aqueous leaf extract

**Fig. 1:**
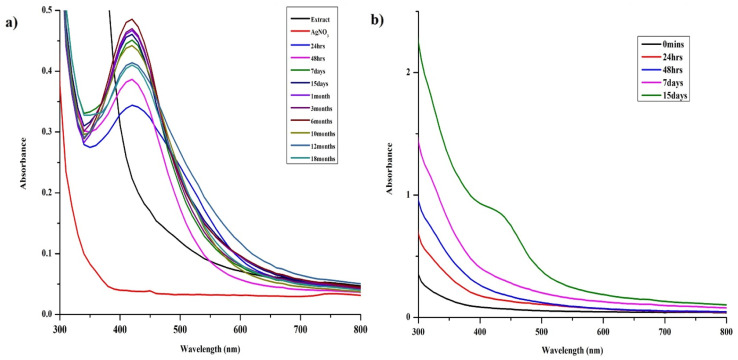
Optimization of silver nanoparticles synthesis using *S. asoca* leaf extract, UV-vis spectra illustrating (a) the synthesis of AgNPs under the light and their stability (reduced mixture stored in dark at room temperature), (b) the synthesis of AgNPs under dark conditions.

AgNPs were prepared by reducing the aqueous AgNO_3_ solution using aqueous leaf extract of *S. asoca* (Roxb.) as described in Materials and methods. The reduction process was accompanied by a color change of the system from golden yellow to reddish brown. Yield of the reduced material corresponding to the AgNPs was confirmed by UV-vis spectrometry. The scanned absorption spectra (300–800 nm) revealed a narrow characteristic surface plasmon resonance (SPR) peak at 420 nm indicating the formation of highly stable and monodispersed particles (Fig. 1a). This characteristic SPR peak that arises due the collective oscillation of the free electrons from the surface of the particles has been used as a primary indication for auditing the synthesis of AgNPs along with the color change.^25,37^ Absorption spectra of the reaction mixture were also recorded at regular time intervals to monitor not only the formation but also the stability of the particles. This, previously described methodology for monitoring of NPs synthesis and stability,^1,27,38^ demonstrated the efficient formation of highly potent and durable AgNPs.

#### 3.1.1 Mechanism of light induced synthesis of AgNPs

**Scheme 1:**
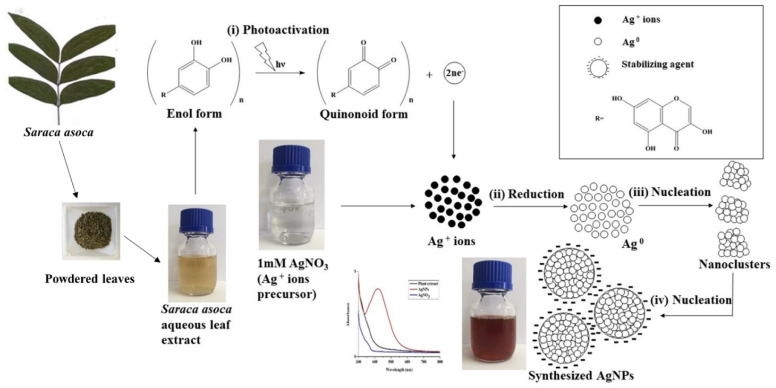
Proposed mechanism for photo-induced AgNPs synthesis from *S. asoca* aqueous leaf extract.

*S. asoca*, being rich in phytochemical constituents,^39,40^ may provide several oxidation and reduction mechanisms for the reduction of Ag^+^ ions to Ag^0^. We found that reduction under the light was the only possible mechanism for AgNPs synthesis (Fig. 1a and b) using *S. asoca* aqueous leaf extract as the reduction reaction performed under the absence of light totally failed in synthesizing the AgNPs. As our primary target was to obtain highly stable AgNPs, we employed controlled illumination of reaction mixture (1140 lx for 48 h at 40 °C) for the synthesis, which eliminates/limits the risk of excess photoactivation that in turn affects the NPs stability.^41^ Based on this, we propose a 4-stage mechanism for the synthesis of AgNPs (Scheme 1).

**Scheme 2:**
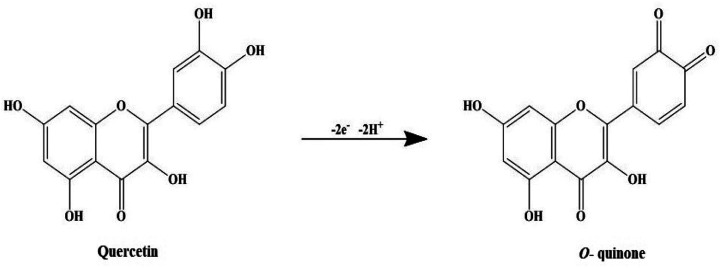
An illustrative model of photo-induced reduction of quercetin's enol form to quinonoid form (*o*-quinone).

**Fig. 2:**
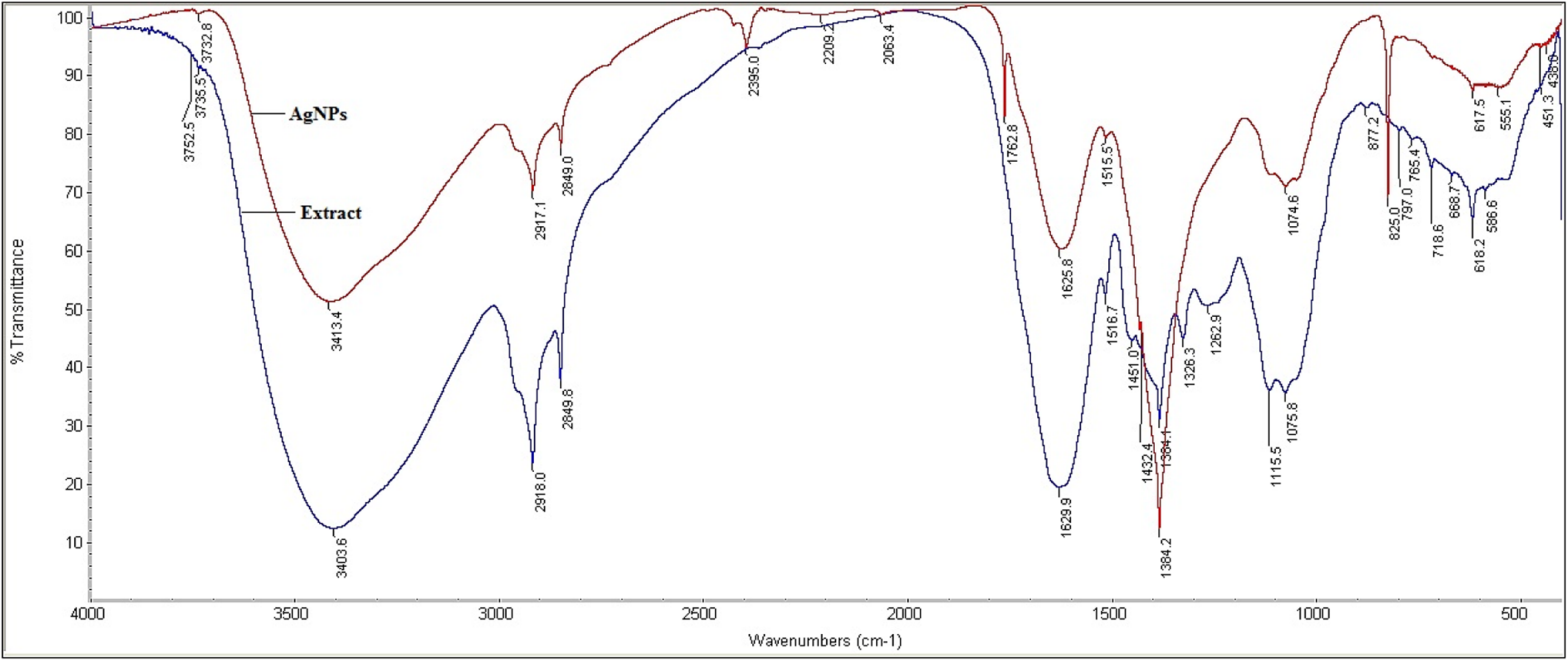
Fourier transform infrared spectrum of *S. asoca* leaf extract (blue) and resulting AgNPs (red).

First stage is photoactivation of the phytochemical constituents (sugars and polyphenolic compounds, especially flavonoids) in the mixture. It has been reported that *S. asoca* leaf extract is rich in quercetin, a photo-active polyphenolic compound (flavonoid),^42^ that can oxidize from its enol to quinonoid upon photo-activation. The oxidation releases two electrons by removing two hydrogen atoms from two hydroxyls as shown in Scheme 2. Second stage is the reduction of silver ions, where the two electrons released during flavonoid oxidation reduce 2Ag^+^ ions to 2Ag^0^ (Scheme 2). Third stage is the nucleation of the reduced Ag^0^ into nanoclusters. Fourth stage is the assembly of NPs and their stabilization. The formed nanoclusters underwent further nucleation to assemble into a single large cluster of AgNPs. In this stage the sugars, proteins and other constituents from the extract bind to the nanoparticles, thus stabilizing them. We confirmed the involvement of sugars, proteins, and flavonoids in stabilization of the particles by FTIR spectrum, showing a strong –OH and –NH_2_ stretch (Fig. 2).

The long-term storage of the NPs took place in reduced extract, which assisted in the maintenance of the repulsive forces between the particles increasing their stability. In many cases (especially chemical and green synthesis), it has been proved that the particles stored in their reaction mixture were more stable than the purified particles. This is due to the removal of stabilizing agent from the surface of the particles during separation (*e.g.*, centrifugation, dialysis) that significantly affects the repulsive force between the particles resulting in aggregation impacting their long-term storage.^1,18,19,21,27^

### 3.2 Optimization of AgNPs synthesis using *S. asoca* leaf extract

Optimization of the synthesis of AgNPs by controlling their size, shape and stability may provide material suitable for high-end industrial applications.^1,36^

#### 3.2.1 Effect of light in AgNPs synthesis and storage

To assess the effect of light on AgNPs synthesis, we carried out UV-vis spectral analysis of the samples exposed to both light and dark, that showed (Fig. 1a and b) the sample processed in dark completely failed to form AgNPs (monitored until 15 days at 40 °C, Fig. 1b). This indicated the importance of light in the synthesis of AgNPs from the aqueous extract of *S. asoca*. Many researchers have reported on the rapid synthesis of AgNPs by exposing the reaction mixture to sunlight, which was triggered by the photo-activation of polyphenolic constituents in the extract.^37,43,44^ This type of synthesis suffers from significant risk of excessive light exposure that further photo catalyzes the components of the mixture serving as the capping agent. This subsequently causes a major change in particle morphology, directly impacting the stability (agglomeration).^45^ Therefore, to address this issue, we used controlled illumination of the mixture with prolonged reaction time at a constant temperature. The sample exposed to fluorescent tube light, readily formed AgNPs. The color change of the reaction mixture from golden yellow to reddish brown indicated the formation of AgNPs yielding a single characteristic SPR (surface plasmon resonance) band at 420 nm.

To evaluate the effect of light on the stability, the reduced mixture was maintained at ambient light 180 lx under normal laboratory conditions (20 ± 3 °C). The NPs precipitated within 15 days resulting in muddy brown solution accompanied by sedimentation of both the extract and particles. The aggregates were subjected to electron microscopy along with EDX analysis, which clearly revealed oxidation of the particles at a higher level (Fig. S1†). Similar observation was reported by researchers who investigated the long-term stability of AgNPs synthesized by green synthesis and chemical methods, where the NPs aggregated when stored under light.^19,27^ The main reason was the reduction of the unconverted/dissolved (leached) Ag^+^ ions to Ag^0^, upon the exposure to light. The Ag^0^ further nucleated the particles that lacks capping that significantly impact their long-term storage. We observed a similar phenomenon with samples stored under light, where, the unconverted Ag^+^ ions from the system were converted to Ag^0^ or the stabilizer of the NPs underwent further reaction resulting in the dissolution of the particles leading to their aggregation. Our observations and the data of other researchers suggest that the suitable conditions for the synthesis of stable AgNPs and their storage are: exposing the reaction mixture to illumination (1140 lx) under a fluorescent lamp for 48 h and dark long-term storage of the whole reaction mixture.

#### 3.2.2 Effect of pH

Many studies have suggested that the pH of the system significantly influences the synthesis of NPs, especially in the aspect of controlling their size and shape. Usually, the best results were obtained with basic pH.^1,46–48^

**Fig. 3:**
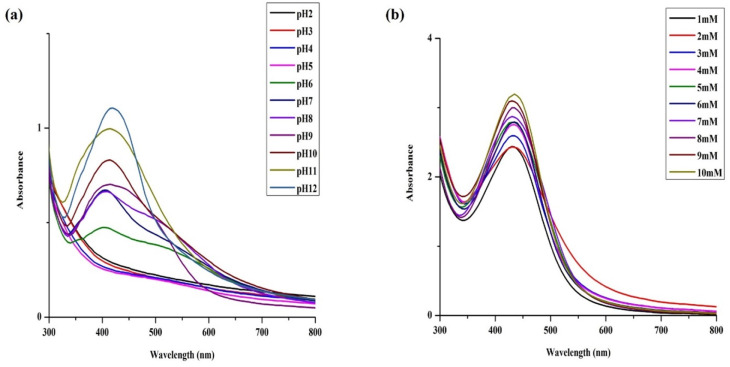
Optimization of silver nanoparticles synthesis using *S. asoca* leaf extract. UV-vis spectra illustrating (a) the effect of pH in the synthesis of AgNPs, (b) the effect of AgNO_3_ concentrations in the synthesis of AgNPs.

AgNPs synthesis reaction performed at acidic pH (ranging from 2 to 5) resulted in immediate precipitation of the reaction mixture thus failing to formed AgNPs. The yield of the reaction improved at pH 6; however, NPs aggregation occurred within 4 days as confirmed by electron microscopy (Fig. S2†). At pH 7, the reaction was fast and provided dark brown product characterized by a single SPR band at 420 nm (Fig. 3a) corresponding to stable AgNPs. Particles prepared at increased basic conditions (pH 8 and 9) exhibited asymmetrical and broad SPR band suggesting their low monodispersity and consequently low stability. In contrast to higher acidic conditions, highly basic pH (10–12) supported fast reaction turning the reaction mixture to reddish dark brown color with symmetrical and narrow SPR band, indicating formation of stable, highly monodispersed NPs. Electron microscopy results were in accord with the above statement. We observed slight agglomeration of the particles at pH 9 and uniformly dispersed particles at pH 12 that started aggregating after a week. As our primary focus deals with the long-term stability, we preferred AgNPs synthesized at pH 7 at which the particles were not subjected to much of the physicochemical changes within a short time (Fig. 1a). Moreover, the studies that investigated on the optimization of the synthesis process have also suggested that the neutral pH being ideal for better stability and biological applications.^1,49^

#### 3.2.3 Effect of silver nitrate concentration on NPs synthesis

Concentrations of AgNO_3_ ranging from 1 to 10 mM, exhibited a satisfactory synthesis rate of particles providing a single narrow SPR band. The intensity of the SPR band increased with increasing concentration of AgNO_3_ representing monodispersed particles in the system. As the primary focus of our work was the stability of AgNPs, the lowest concentration (1 mM AgNO_3_) was chosen as optimal due to its ability to exhibit long-term stability with very good monodispersity (Fig. 3b). The higher AgNO_3_ concentrations (>5 mM) were also suitable for production of monodispersed AgNPs (within 48 h), but the particles aggregated within five days. Large particles eventually settled down at the bottom of the test tube within eight days. Our observations were consistent with previous studies that revealed that the concentration of AgNO_3_ plays a major role in the yield of monodispersed AgNPs. It provided optimal results at low concentrations but failed to stabilize at higher concentrations.^1,49^

#### 3.2.4 Effect of the ratio of *S. asoca* leaf extract to AgNO_3_ in the synthesis of AgNPs

**Fig. 4:**
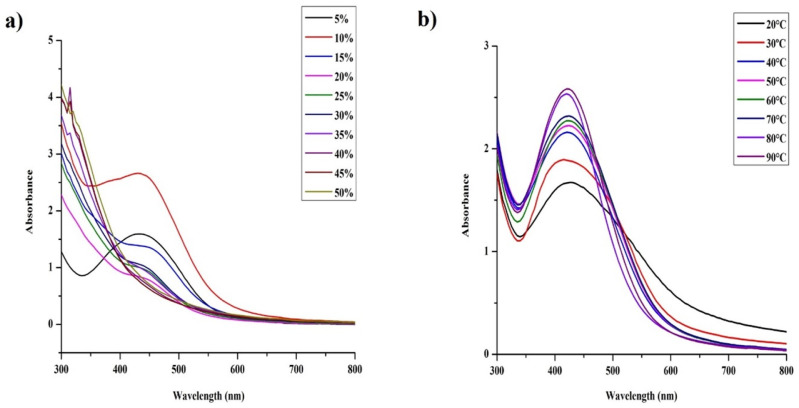
Optimization of silver nanoparticles synthesis using *S. asoca* leaf extract, UV-vis spectra illustrating (a) the effect of different proportions of *S. asoca* leaf extract to the different proportions of 1 mM AgNO_3_ in the synthesis of AgNPs, and (b) the effect of temperature in the synthesis of AgNPs.

Numerous studies have demonstrated the importance of extract concentration playing a crucial role in the acceleration of AgNPs synthesis.^1,44,50^ One of the previous reports that employed dark synthesis for AgNPs production, documented a synchronous increase in the reaction rate and the yield of highly monodispersed AgNPs with an increase in the ratio of the extract to metal salt.^1^ However, the same team reported that particles synthesized with a higher extract volume ratio are unstable and not suitable for studying their long-term stability. On the contrary, in our synthesis method, we observed a drop-in reaction rate at higher volume ratios (>15%, Fig. 4a) of the extract leading to NPs synthesis after three days (Fig. S3†) and at very high ratios (>30%) a total drop-in the reaction rate was noted, resulting in complete failure of particle synthesis. Our observations were in accord with the previous studies that employed sunlight induced synthesis of AgNPs, where the authors suggested the optimization of extract ratio in the mixture along with the sunlight exposure time to solve this issue.^25,36,37,51^

To further study the effect of proportion of the extract in the reaction mixture, we combined different proportions of the extract with different concentrations of AgNO_3_ (namely 1, 3, 5, 7, 10 Mm). We found that the increase of the AgNO_3_ concentration facilitates effective synthesis of AgNPs even at the higher proportions of the extract within 48 h (Fig S4d and e†). In contrast, higher concentration of the extract completely failed to synthesize the AgNPs at the lower salt concentrations even after 3 days of synthesis (Fig. S3†). As discussed above, the lower salt concentrations performed well in the synthesis of AgNPs with lower concentrations of extracts (1–5 mM, Fig. S4a–c†) unlike the higher concentrations, where they totally failed in the synthesis. Concurrently, the higher salt concentrations did perform well with the higher salt concentrations (7 and 10 mM) (Fig. S4d and e†) in the synthesis of AgNPs. In case of highest salt concentration (10 mM), we were able to observe the synthesis of monodispersed AgNPs resulting in a single narrow SPR at 420 nm even at 50% of the extract in the mixture within 48 h, which wasn't possible to be achieved at the lower concentrations. Moreover, with increasing AgNO_3_ concentration, the lower proportions of the extract did suffer from improper reduction resulting to the formation of bigger particles consequently exhibiting a wide SPR peak (especially observed with 7 and 10 mM, Fig. S4d and e†).

To summarize from the observations above, we could clearly see the possible scope for bulk synthesis of AgNPs using our method, but still other parameters such as the intensity of the light, exposure duration, temperature need to be considered.

As we can see in section 3.2.3, particles synthesized with higher concentration of AgNO_3_ did exhibit low stability, thus to achieve the long-term stability of AgNPs, lower concentration of AgNO_3_ was preferred. From above, we could conclude that the lowest salt concentration with lower extract proportion did facilitate the faster synthesis of stable AgNPs when compared with other concentrations of the extract and the salt. Furthermore, considering the reaction rate, the AgNPs yield and the risk of prolonged light exposure, we found 10% (v/v, 4 g l^−1^) of the *S. asoca* extract in 90% 1 mM of AgNO_3_ with 48 h of light exposure to be suitable for the NPs synthesis.

UV-vis spectra illustrating (a) the effect of different proportions of *S. asoca* leaf extract to the different proportions of 1 mM AgNO_3_ in the synthesis of AgNPs, and (b) the effect of temperature in the synthesis of AgNPs.

#### 3.2.5 Effect of temperature on NPs synthesis and storage

Many studies have shown that the temperature plays a vital role in accelerating the formation of AgNPs.^1,52^ Similarly, while evaluating the effect of light in the synthesis of AgNPs, we also found that 40 °C supported faster synthesis of NPs than compared to the room temperature (Fig. 4b). However, the samples incubated at 40 °C in dark completely failed to produce AgNPs (Fig. 1b). Although temperature is an important factor in the light-induced production of AgNPs and elevated temperatures above 40 °C have further increased the synthesis rate (Fig. 4b), the risk of oxidation ultimately leading to AgNPs aggregation forced us to avoid higher temperatures. The particles synthesized at 30 °C and 20 °C exhibited a wide and significantly irregular SPR band indicating that the low reaction rate resulted in the formation of large particles. Due to a high synthesis rate at 40 °C, this temperature was chosen as optimal for the synthesis of AgNPs. The resulting particles were highly stable and monodisperse and showed a narrow SPR band at 420 nm. Although, during the synthesis, higher temperature accelerates the reaction rate, the particles stored at low temperatures usually exhibit better stability. It has been reported that the particles storage temperature directly influence physicochemical changes such as increased ion release and oxidation that triggers morphological changes in the system leading to particles aggregation.^18,19,27^ The authors also proved that particles stored in the dark at 4 °C are extremely stable and exhibit less physicochemical changes when compared to particles stored at room temperature. The dark storage at room temperature is commonly reported storage method of green synthesized AgNPs.^1,24,27,47^ And as our main aim was to study the antimicrobial durability of green synthesized AgNPs, we used the particles stored in dark at room temperature (20 ± 3 °C) as they have been known to undergo comparatively slower physiochemical changes than the particles stored under light or at higher temperature^19^ for further studies.

### 3.3 Characterization of freshly synthesized AgNPs

#### 3.3.1 Morphological TEM analysis

**Fig. 5:**
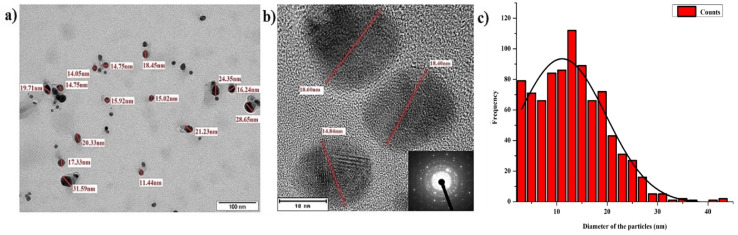
Characterization of fresh (48 hours old) silver nanoparticles synthetized using *S. asoca* leaf extract (a) TEM image of spherical nanoparticles with the size of 10–25 nm; scale bar is 100 nm, (b) HR-TEM image of spherical nanoparticles; scale bar is 10 nm, inset shows the SAED pattern of silver, (c) histogram analysis of particle size distribution of AgNPs from TEM measurements (histogram was generated by analyzing multiple images acquired from the freshly synthesized AgNPs).

As reported by many researchers, the size range and shape of the AgNPs can be optimized by altering the pH and temperature of the reaction system.^46,53^ The shape and size of the synthesized AgNPs was determined by conventional TEM. Most of the particles were spherical (Fig. 5a and b) with most of the particle sizes lying between the range of 5–20 nm (Fig. 5c) and with the average size distribution of 11.1 ± 8.7 nm, suggesting that our optimization process was successful.

#### 3.3.2 FTIR analysis

As reported earlier, phytochemical constituents in the reaction mixture (AgNO_3_ + plant extract or cell free filtrate) play a vital role in the synthesis of AgNPs.^1,47,54^ In our study, we employed Fourier transform infrared spectroscopy (FTIR) for the qualitative analysis of functional groups attached to the synthesized NPs. This analysis was done by comparing the spectrum of leaf extract to the spectrum of synthesized AgNPs. Both spectra shown in Fig. 2 were evaluated according to the previous reports.^50,55,56^ The peaks at 718, 765, 797 and 825 cm^−1^ correspond to aromatic C–H stretch, the peaks 1516 and 1625 cm^−1^ correspond to –NH (amide II) and (amide I) stretching respectively. The strong bands observed at 1384 and 1629 cm^−1^ in the spectra correspond to C–N or C–O and C=C stretching of the carboxylic group. The stretch 3403 and 3413 cm^−1^ correspond to phenolic O–H and –NH_2_ stretch. The peaks 2849 and 2918 cm^−1^ correspond to alkyl C–H stretch, 1625 cm^−1^ peak corresponds to aryl conjugate C=C stretch, 1762 cm^−1^ peak corresponds to ketone C=O stretch. To conclude, our FTIR results confirmed the presence of –NH, –OH, C=C, and –CH groups, indicating the involvement of the hydroxyl and amine groups in the synthesis and stabilization of AgNPs. We therefore assume that flavonoids present in the *S. asoca* aqueous leaf extract^57^ are responsible for the reduction of Ag^+^ ions to Ag^0^ facilitated by photoactivation. The sugars, amines and other compounds present in the extract facilitated the capping of synthesized particles. Thus, our FTIR results confirmed the ability of the *S. asoca* aqueous leaf extract to both reduce and stabilize the synthesized AgNPs.

### 3.4 Physicochemical changes experienced during long-term storage of AgNPs

#### 3.4.1 Morphological changes during long-term storage of AgNPs

**Fig. 6:**
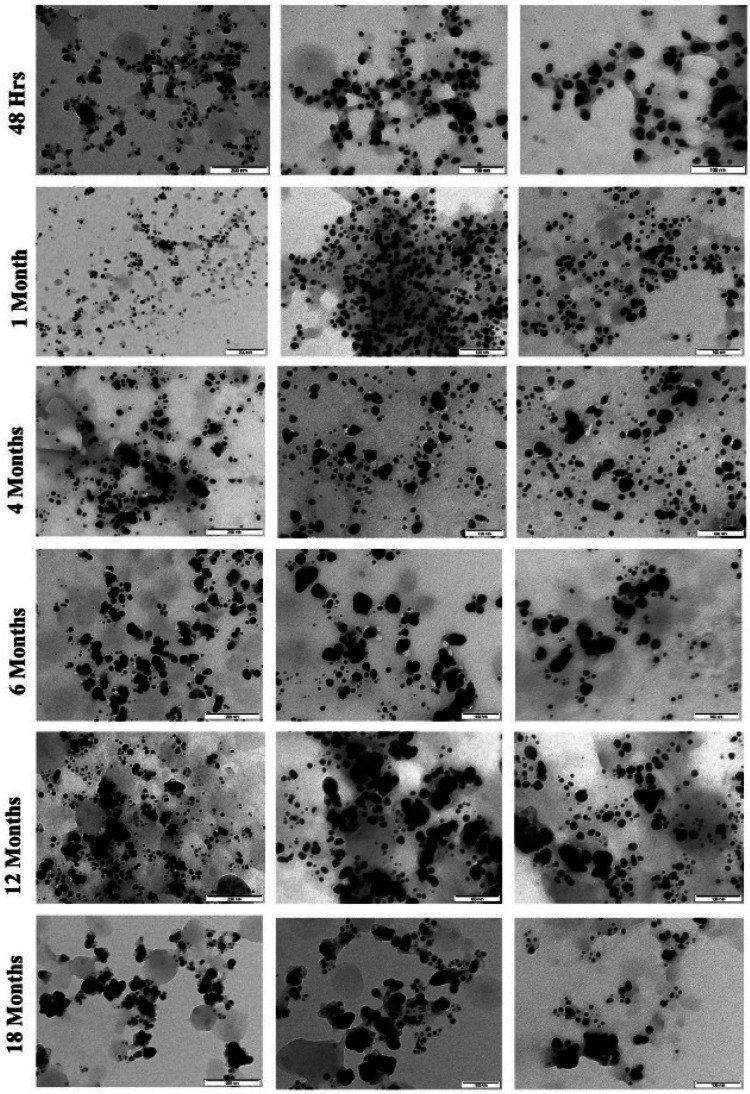
TEM images of AgNPs synthesized by controlled illumination and stored in dark conditions at room temperature illustrating the effect of aging at different time intervals.

The morphological changes that the particles encounter during long-term storage was mainly caused by the storage conditions. Usually, particles stored in the dark at very low temperature are extremely durable with much less physicochemical changes regardless of the synthesis and stabilization methods used.^18,19,27^ As we observed physicochemical changes that affect antimicrobial activity during long-term storage of AgNPs, we employed dark storage at room temperature as the only storage condition, as it is known for undergoing much less physicochemical changes than that stored under light (section 3.2.1). Similar failure of stabilizing agent during light exposure of green synthesized AgNPs was published previously.^27^ In our study, slow synthesis under controlled illumination with prolonged light exposure at constant temperature (1140 lx for 48 h at 40 °C) of the reaction mixture provided very good yield of stable AgNPs. The product provided a single SPR band at 420 nm (after 48 h) representing majority of particles of a particular size range (Fig. 1a). TEM evaluation of the particles also revealed uniform size of the particles (Fig. 5c), where more than 80% of the particles fell into the size range of 5–20 nm with average size falling around 11.1 ± 8.7 nm. The AgNPs also exhibited very good stability in dark storage at room temperature throughout the study with no significant change in the absorption spectra (Fig. 1a). During the seventh day, a shift in SPR band from 420 nm to 425 nm was noticed without widening of the SPR band suggesting no change in monodispersity. During the sixth month after the synthesis, slight widening of the SPR band was noticed suggesting the increase in NPs polydispersity (agglomeration), and the TEM analysis of the sample also proved this. The TEM analysis of 4- and 6 months old sample revealed slight aggregation (Fig. 6). Interestingly, there were no major differences in SPR band shift until 6th month; but since the sixth month, we noticed the broadening of the SPR spectrum, and the highest broadening was observed with the 10-, 12- and 18- months old samples suggesting the highest rate of aggregation, accompanied with the fall in the intensity of the absorbance. The ageing of the particles is observed as a gradual color change of the system from dark brown to dark reddish brown indicating a slow loss of stabilizing effect of the extract. Despite this, no deposition was observed till 12th month, but slight deposition was observed after 12th month and with 18 month-old particles very thick layer of deposition was observed. Accordingly, TEM evaluation of the 12 and 18 months old sample showed high signs of aggregation (Fig. 6) accompanied by very thick patches of extract binding the particles (especially 18 months old particles). This aggregation could be caused by the degradation of the stabilizing agent (in our case the plant extract components) from the particles surface, which subsequently reduced the repulsive forces between the particles.

**Fig. 7:**
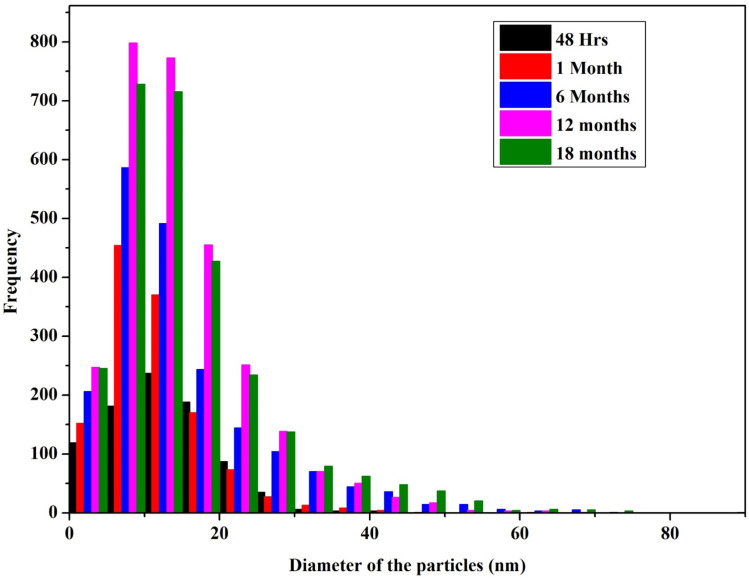
Histogram of the particle size distribution of AgNPs (stored in dark conditions at room temperature) measured using TEM at different time intervals (an average of 10–12 high resolution images from each sample was considered for the size analysis).

The TEM analysis (Fig. 7) at different time intervals (48 h, 1 month, 6 months, 12 months, and 18 months) revealed increased size distribution of the NPs with their age. Freshly synthesized particles had a size range of 5–30 nm while the 12 months and 18 months old particles range within 5–70 nm and 5–90 nm respectively. The varied size range of the old particles could be due to the formation of aggregates caused by the failure of the stabilizing agent. We also observed that the old nanoparticles (6, 12 and 18 months) had the highest frequency of smaller particles between the range of 5–20 nm, likely due to the nucleation of new particles by Ag^+^ ions leached from the old particles. Similar observation was previously reported with the particles stored in dark at room temperature, where the authors described the formation of new particles as a result of nucleation of Ag^0^ that was reduced from the dissolved Ag^+^ ions.^27^ Interestingly, we observed most of the particles in the size range of 5–30 nm, which could be the main reason maintaining their antimicrobial activity even at the high rate of aggregation of aged AgNPs.

#### 3.4.2 Oxidation and ion release during long-term storage of AgNPs

**Fig. 8:**
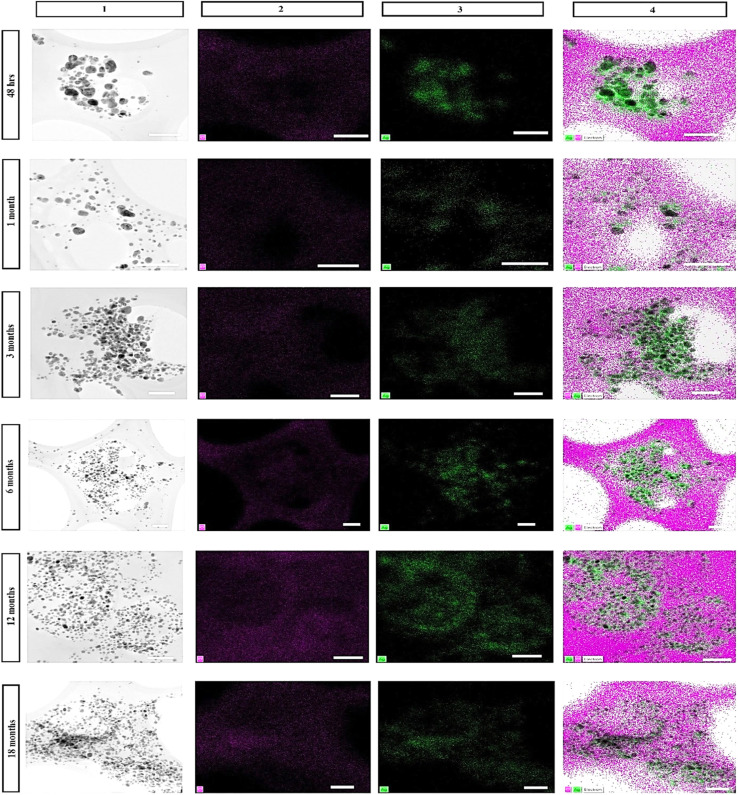
HR-TEM images with EDS elemental mapping of AgNPs stored under dark conditions at room temperature at different time intervals (scale bar 100 nm): (1) EM image, (2) single element mapping of oxygen, (3) single element mapping of silver corresponding to the EM image, (4) combined chemical mapping of EM image, oxygen, and silver.

**Fig. 9:**
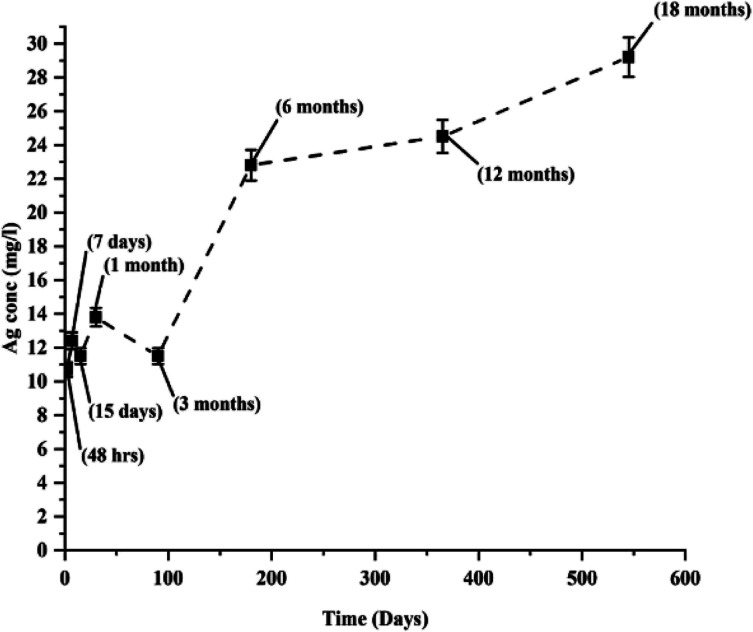
AAS measurement of Ag^+^ ions released from AgNPs during their long-term storage with the reduced mixture.

To analyze the elemental composition, the particles were subjected to EDX elemental analysis. The scanned beam (transmitted electron beam) created the map of elemental composition of the particles. The scanned images showed the obvious presence of silver and other elements such as oxygen, carbon, and chlorine as major constituents; and silicon, sulphur, and potassium as minor constituents. The carbon originated from the grid as well as from the sample (extract + AgNPs) and oxygen from the reduced extract as well as from the particles. The oxygen signals throughout the image resulted from oxidized extract in which the NPs were stored. However, oxygen signals from the particles could be clearly differentiated from the signals from the extract based on their elevated intensity in the regions of the particles in the elemental analysis map (Fig. 8). Oxidation of AgNPs lead to their partial dissolution resulting in Ag^+^ ions release from the particles. This phenomenon is considered as an important characteristic of AgNPs toxicity. Generally, oxidation of AgNPs depends on the storage conditions and stabilizing agent. Many researchers reported that the particles stored under the light underwent a significant oxidation resulting in partial dissolution of the particles accompanied with a change in their morphology. This happened due to the failure or photo-activation of the stabilizing material. To address this issue, high molecular weight stabilizers (Tween 80® and Disperbyk-129) were employed, which totally engulf the particles in turn reducing oxidation but increasing the hydrodynamic size impacting their biological applications. High molecular weight stabilizers also exhibit toxicity at higher concentrations.^18–20,27^ We also found that the particles formed large aggregates increasing their polydispersity due to photocatalytic change of the stabilizing components in the extract and synthesis of new particles from the dissolved/unconverted Ag^+^ ions. The elemental analysis of the aggregates disclosed significant oxidation of the particles (Fig. S1†). However, the particles stored in dark did not reveal any morphological change and traces of oxidation until 6 months after synthesis. After the 6th month, the samples became gradually oxidized (Fig. 8). EDX point analysis of the individual particles supported this finding (Fig. S5†). Despite very strong stabilizing effect of the extract, low open-air exposure, and storage in the dark; the particles oxidized. This could be due to the degradation of stabilizing components at the surface of AgNPs. Measurement of the Ag^+^ ions release from the particles (Fig. 9) supported the statement above, where the 6 month old particles released two times more ions than those initial (48 h) particles. This type of increase in Ag^+^ ion has been previously reported,^19^ where the team has employed different categories of stabilizers (sodium citrate, SDS, chitosan, Tween 80® and Disperbyk-129) to stabilize chemically synthesized AgNPs. More of the ion release was observed with the particles stabilized with the ionic stabilizers than the high molecular weight stabilizers. Similarly, in our study we also found the increase in ion release with the age, but a drastic – two fold change in the ionic concentration, was not noticed in our work after the first 6 months proving majority of the changes in the stability occurring within this time. In addition, it is worth of noting that the first sign of high oxidation appeared in the 6 months old sample.

### 3.5 Antimicrobial durability of AgNPs

**Fig. 10:**
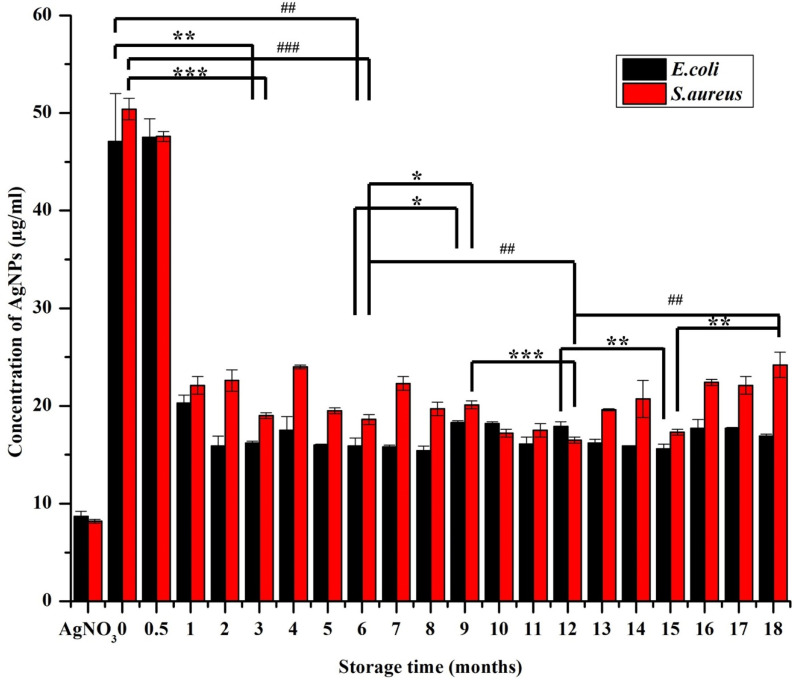
Antimicrobial ability (IC_50_) of AgNPs (synthetized using *S. asoca* leaf extract stored in dark at room temperature) monitored at regular time interval of every 30 days, data are presented as mean (*n* = 3) ± SEM, the comparison of the data between every 3 months is represented as *p ≤ 0.05, **p ≤ 0.01, ***p ≤ 0.001, and between every 6 months is represented as ^#^*p* ≤ 0.05, ^##^*p* ≤ 0.01, ^###^*p* ≤ 0.001 significance level.

**Fig. 11:**
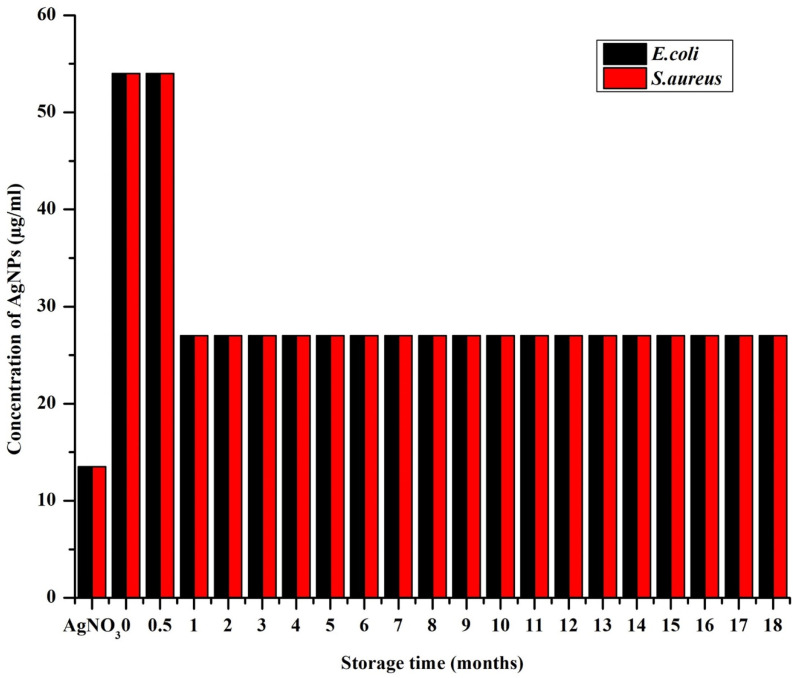
Minimum inhibitory concentration (MIC) of AgNPs (synthetized using *S. asoca* leaf extract stored in dark at room temperature) monitored at regular time interval of every 30 days, data are presented as mean ± SEM, *n* = 3.

*In vitro* antimicrobial activity of the AgNPs was determined by broth dilution assay, which was documented as IC_50_ and MIC (Fig. 10and 11, respectively). The freshly synthesized AgNPs (48 h old) were almost nontoxic due to the superior stabilizing effect of the extract that prevented the AgNPs from releasing the ions. Specifically, they exhibited the lowest rate of inhibition with the IC_50_ values of 47.1 ± 4.9 μg ml^−1^ and 50.4 ± 1.2 μg ml^−1^ for *E. coli* and *S. aureus*, respectively and MIC at 54 μg ml^−1^ for both *E. coli* and *S. aureus*, where the starting concentration for the analysis was 54 μg ml^−1^. Interestingly, the inhibition rate increased by more than half within the first month resulting in the IC_50_ values of 20.3 ± 0.8 μg ml^−1^ and 22.1 ± 0.9 μg ml^−1^ for *E. coli* and *S. aureus*, respectively. After this MIC drop to 27 μg ml^−1^ for both *E. coli* and *S. aureus*, it remained the same throughout the whole study period. This could be because the slow dissolution of the particles. The highest inhibition of *E. coli* was exhibited by AgNPs stored from three up to eight months where the IC_50_ ranged between 15.4–15.9 μg ml^−1^. After 10 months, the IC_50_ increased to 18.3 ± 0.2 μg ml^−1^ and through the rest of study period the IC_50_ values ranged between 16.2 and 18.3 μg ml^−1^ (Fig. 10). With *S. aureus*, the highest inhibition was recorded between 10 and 12 months of NPs storage with IC_50_ between 16.5 and 17.5 μg ml^−1^. A significant fall in the inhibition rate was observed with the NPs stored over 12 months. These observations were in accord with the observations of a previous report dealing with the effect of different storage conditions (dark, light, cold storage) of the laser generated AgNPs on their antibacterial activity.^18^ In that study, the freshly made particles exhibited much less inhibition and as the particles aged their inhibition ability increased and towards at the end of the study (within 10 months) there was a gradual and total drop in inhibition (similar pattern in inhibition rate was observed with *S. aureus* in our study). This type of change in antimicrobial activity was mainly because of the physicochemical changes that occurred with time during storage such as frequent air and light exposure. Similarly, in the studies of the long-term toxicity of chemically stabilized AgNPs stored with different kind stabilizing agents, the particles stabilized with high molecular weight stabilizer (Tween, Disperbyk and Byk) exhibited very low toxicity in the beginning that changed with the time.^19,20^ These particles also exhibited better stability when compared to other commonly employed stabilizing agents (ionic stabilizers such as SDS, citrate, chitosan) that undergo higher physicochemical changes. To avoid this type of fluctuations in toxicity, we thoroughly optimized the storage conditions of our particles and achieved consistent long-term antimicrobial activity throughout the study period. This observation was due to the strong stabilizing effect provided by the extract along with the storage conditions that prevented the change of physicochemical properties of our particles. Notably, we noticed *E. coli* exhibiting slightly higher (non-significant in most of the cases) sensitivity towards AgNPs when compared to *S. aureus*. The difference in inhibitory activity of AgNPs of the microorganisms was mainly due to different structure and chemical composition of the cell wall of the representatives of Gram-positive and Gram-negative bacteria (Fig. 10).^14,58,59^

## 4 Conclusions

To the best of our knowledge, our study is the first to analyze the periodic changes in the physicochemical properties of green synthesized AgNPs that influenced their antimicrobial durability. We have introduced a new method for the green synthesis of AgNPs which is based on light induced slow synthesis with a prolonged exposure to a controlled light source at a constant temperature. The main advantage of this method is prevention of under/over exposure of the components that assists in the synthesis and capping of AgNPs that significantly affects the long-term stability, which is a major issue in conventional light induced methods employing high intensity light source (generally sun light), that facilitates a very rapid synthesis that often results in under/over exposure.

We optimized and validated the parameters that played a major role in synthesis and stability of AgNPs. From these, the light exposure time and extract volume in the system were decisive for effective synthesis. The stability data presented here clearly state that the changes in AgNPs' physicochemical properties (especially oxidation) during their long-term storage were responsible for their variable time dependent toxicity. The AgNPs synthesized by our method did exhibit varying antimicrobial ability both in the case of *E. coli* and *S. aureus* throughout the study period of 18 months. Notably, the NPs did not lose their antimicrobial ability toward the end of the study despite the huge physicochemical changes experienced by the NPs during their long-term storage. Although achieving size uniformity of AgNPs during the synthesis is quite demanding by green synthetic methods when compared with the conventional physicochemical methods, it is achievable by optimization the reaction parameters that would ultimately also affect the NPs stability. We believe that our study provides useful insights into the processes accompanying the ageing of AgNPs and gives us basic information that to help improve green synthesis methods.

## Author contributions

Conceptualization, H. G., J. V., and T. R.; methodology, H. G., J. V., and T. R.; formal analysis, H. G.; investigation and validation, H. G., J. V. and P. U. (microscopy); resources, J. V., and T. R.; writing—original draft preparation, H. G.; writing – review and editing, J. V., P. U., and T. R.; visualization, H. G. and J. V.; supervision, J. V. and T. R.; project administration, T. R.; funding acquisition, T. R. All authors have read and agreed to the current version of the manuscript.

## Conflicts of interest

The authors declare no conflict of interest in this study.

## Supplementary Material

RA-012-D2RA04667A-s001
