# Supplementary material for: Effect of the physicochemical changes in the antimicrobial durability of green synthesized silver nanoparticles during their long-term storage
Source: RSC Adv. 2022 Oct 25;12(47):30386–403. doi: 10.1039/d2ra04667a (PMC9594854; doi:10.1039/d2ra04667a)
Supplement: RA-012-D2RA04667A-s001 [file RA-012-D2RA04667A-s001.pdf]

Electronic Supplementary Material (ESI) for RSC Advances.
This journal is © The Royal Society of Chemistry 2022

Electronic supporting information

Effect of the physicochemical changes in the antimicrobial durability of the green synthesized silver nanoparticles during their long-term storage

Giyaullah Habibullah*, Jitka Viktorova, Pavel Ulbrich, Tomas Ruml

Department of Biochemistry and Microbiology, University of Chemistry and Technology, Technická 3, 166 28, Prague, Czech Republic.

***Correspondence: habibuli@vscht.cz**

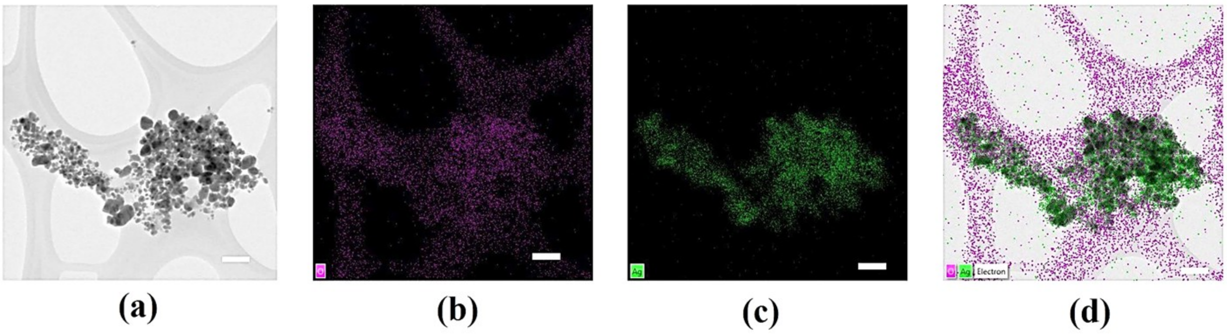

Fig. S1 HR-TEM images (Scale bar 100 nm) of AgNPs stored under light for 15 days; (a) EM image, single element mapping of oxygen (b) and silver (c) corresponding to the EM image, (d) combined chemical mapping of EM image, oxygen, and silver.

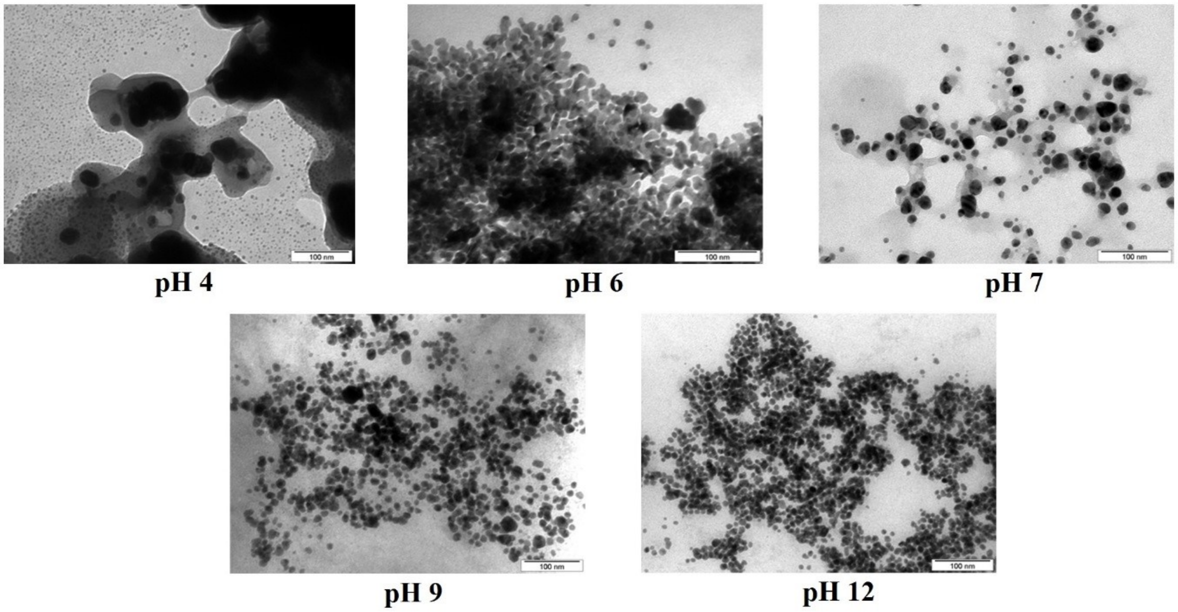

Fig. S2 TEM images (Scale bar 100 nm) of AgNPs synthesized at different pH values.

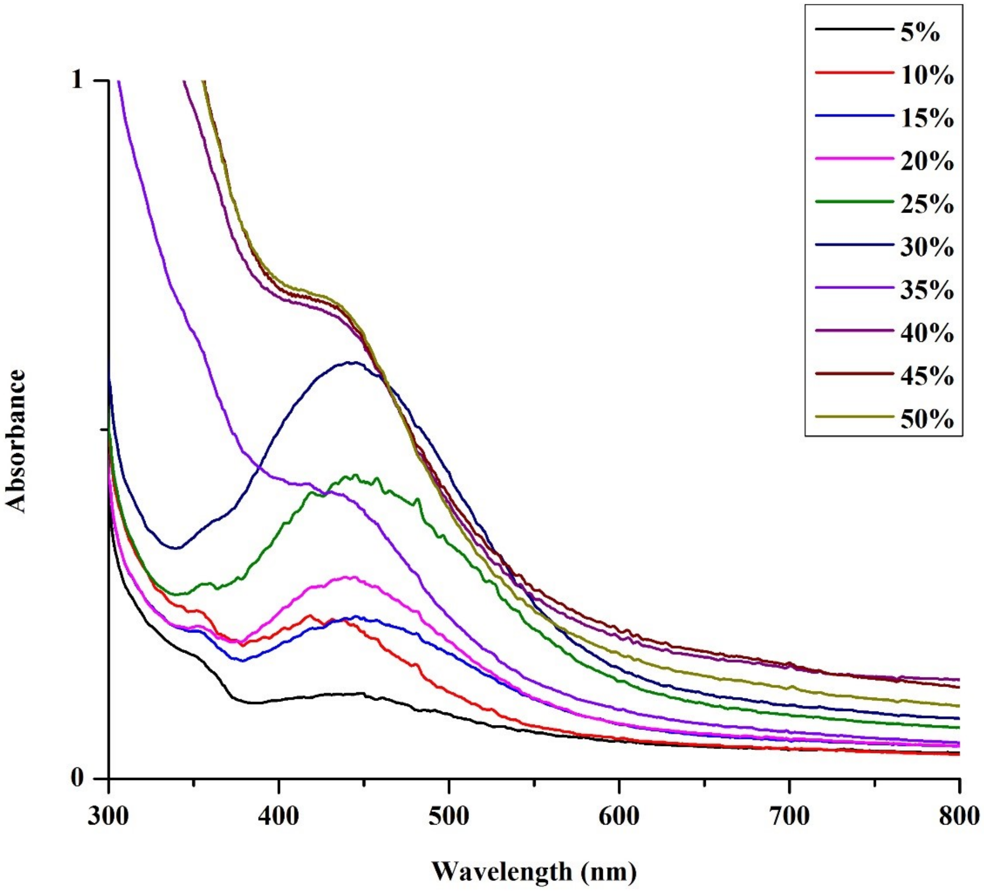

Fig. S3 Optimization of silver nanoparticles synthesis using ***Saraca asoca*** leaf extract, UV-Vis spectrum of AgNPs that illustrates the effect of different volumes ratios of ***S. asoca*** leaf extract (40 g/l) to 1mM $AgNO_3$ in the synthesis of AgNPs on the 4th day.

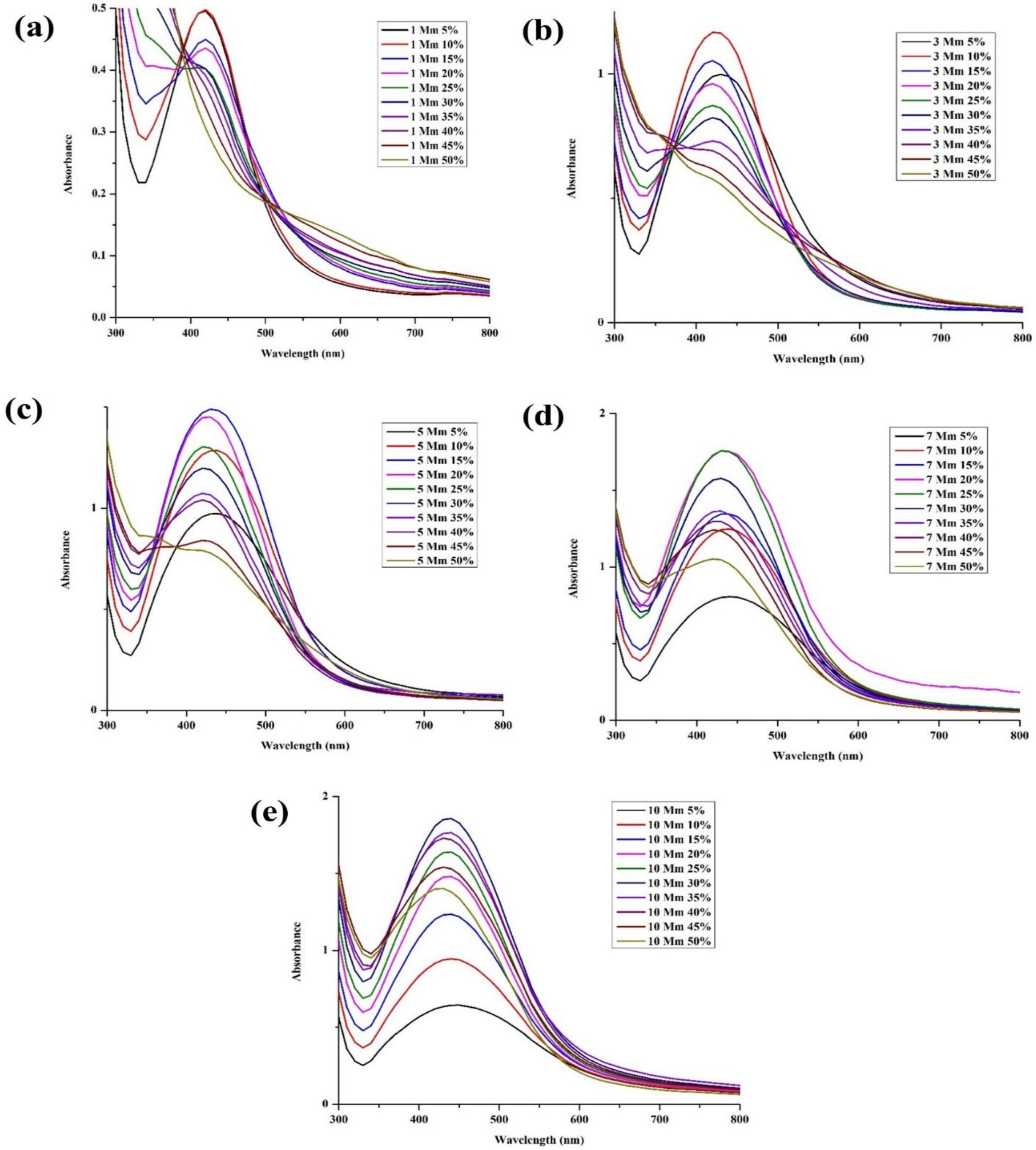

Fig. S4 Optimization of silver nanoparticles synthesis using ***Saraca asoca*** leaf extract,

UV-Vis spectrum of AgNPs that illustrates the effect of different volumes ratios of ***S. asoca*** leaf extract (40 g/l) to (a) 1mM $AgNO_3$ (b) 3mM $AgNO_3$ (c) 5mM $AgNO_3$ (d) 7mM $AgNO_3$(e) 10mM $AgNO_3$ in the synthesis of AgNPs.

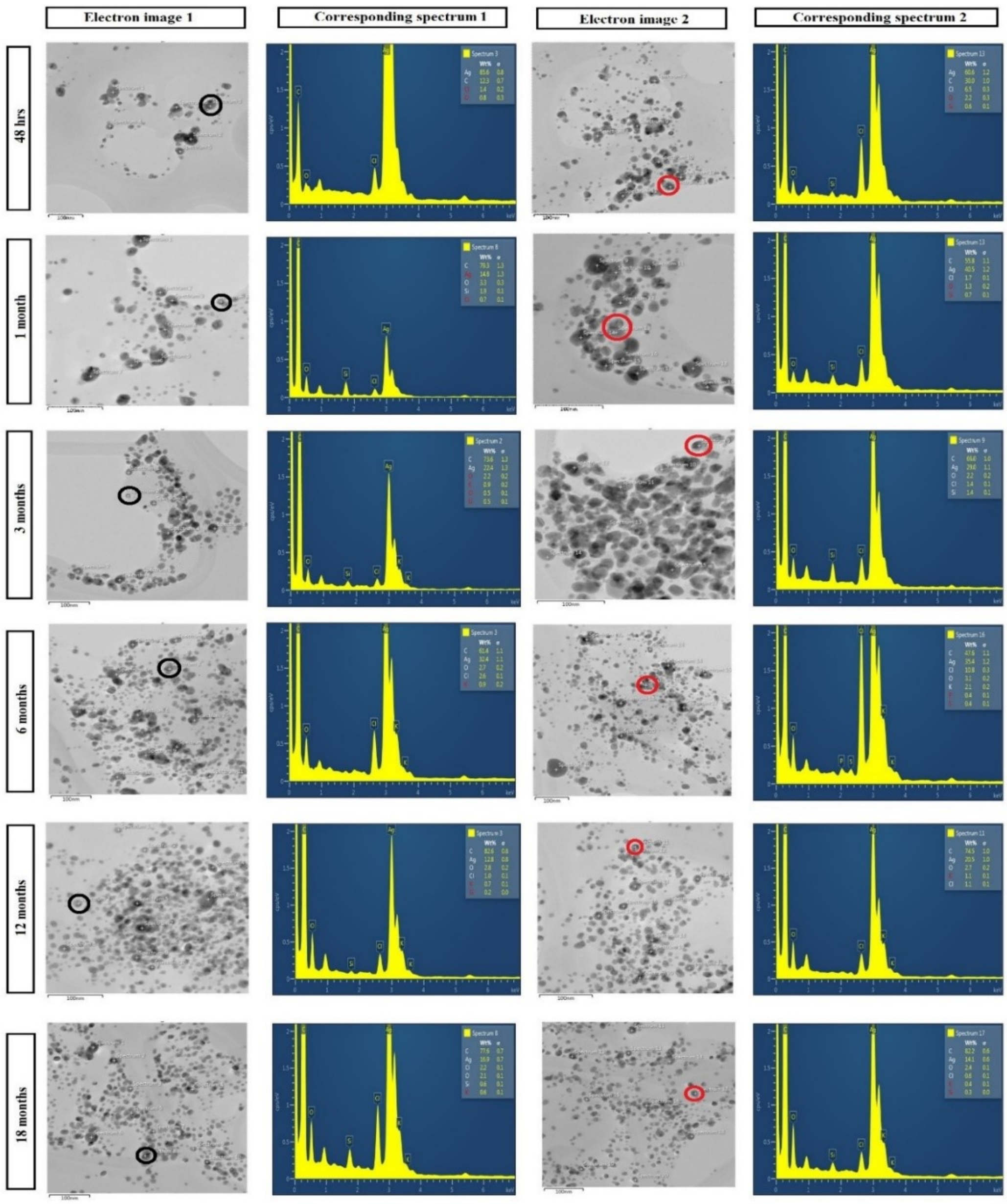

Fig. S5 EDX point analysis of AgNPs stored in dark conditions at room temperature in different time intervals (scale bar 100 nm); the black circle in the electron images 1 refers to the region of origin of the corresponding spectrum 1, the red circle in the electron images 2 refers to the region of origin of the corresponding spectrum 2.
